# 1,4-Dimethyl-2-phenyl-6,7-dihydro-1*H*-pyrazolo­[4,3-*b*]pyridine-3,5(2*H*,4*H*)-dione

**DOI:** 10.1107/S1600536811036233

**Published:** 2011-09-14

**Authors:** Marc Weisser, Dieter Schollmeyer, Stefan Laufer

**Affiliations:** a Eberhard-Karls-University Tübingen, Auf der Morgenstelle 8, 72076 Tübingen, Germany; b University Mainz, Institut of Organic Chemistry, Duesbergweg 10-14, 55099 Mainz, Germany

## Abstract

The mean plane of the pyrazolone ring [maximum deviation = 0.054 (1) Å] of the title compound, C_14_H_15_N_3_O_2_, is oriented at a dihedral angle of 36.05 (7)° with respect to the phenyl ring. The methyl group is slightly disposed [distance = 0.864 (2) Å] out of the mean plane of the pyrazolone ring to which it is attached.

## Related literature

For the biological activity of pyrazolone derivates (*e.g.* dipyrone), see: Pierre *et al.* (2007 ▶). For general methods of clevage of N-Cbz protected amines see: Greene & Wuts (1999 ▶). For conversion of N-Cbz-protected amines into N-*t*-Boc-protected amines, see: Sakaitani *et al.* (1988 ▶).
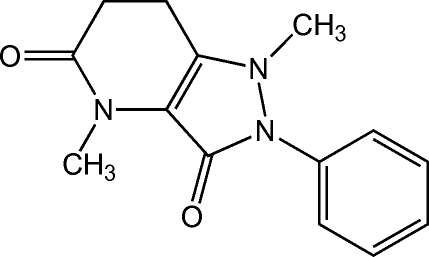


## Experimental

### 

#### Crystal data

C_14_H_15_N_3_O_2_
*M* *_r_* = 257.29
Monoclinic, 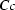

*a* = 8.9721 (7) Å
*b* = 21.7653 (19) Å
*c* = 7.3725 (5) Å
β = 120.214 (5)°
*V* = 1244.12 (17) Å^3^
*Z* = 4
Mo *K*α radiation
μ = 0.10 mm^−1^
*T* = 193 K
0.45 × 0.17 × 0.16 mm

#### Data collection

Stoe IPDS 2T diffractometer
4103 measured reflections
1623 independent reflections
1541 reflections with *I* > 2σ(*I*)
*R*_int_ = 0.036

#### Refinement

*R*[*F* ^2^ > 2σ(*F* ^2^)] = 0.027
*wR*(*F* ^2^) = 0.071
*S* = 1.04
1623 reflections
174 parameters
2 restraints
H-atom parameters constrained
Δρ_max_ = 0.18 e Å^−3^
Δρ_min_ = −0.14 e Å^−3^

### 

Data collection: *X-AREA* (Stoe, 2010 ▶); cell refinement: *X-AREA*; data reduction: *X-RED* (Stoe, 2010 ▶); program(s) used to solve structure: *SIR97* (Altomare *et al.*, 1999 ▶); program(s) used to refine structure: *SHELXL97* (Sheldrick, 2008 ▶); molecular graphics: *PLATON* (Spek, 2009 ▶); software used to prepare material for publication: *PLATON*.

## Supplementary Material

Crystal structure: contains datablock(s) I, global. DOI: 10.1107/S1600536811036233/bt5636sup1.cif

Structure factors: contains datablock(s) I. DOI: 10.1107/S1600536811036233/bt5636Isup2.hkl

Supplementary material file. DOI: 10.1107/S1600536811036233/bt5636Isup3.cdx

Supplementary material file. DOI: 10.1107/S1600536811036233/bt5636Isup4.cml

Additional supplementary materials:  crystallographic information; 3D view; checkCIF report

## Figures and Tables

**Fig. 1.**
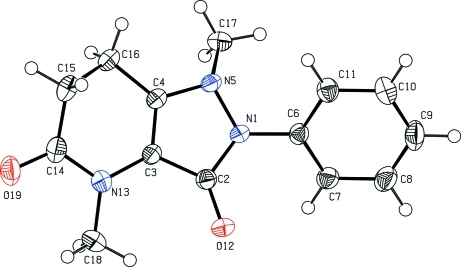
View of compound I. Displacement ellipsoids are drawn at the 50% probability level.

## supplementary crystallographic information

### Comment

Pyrazolone derivates are widely known as potent analgesic drugs (Pierre *et al.*, 2007). By transforming the protection group from a N-Cbz group into a N-*t*-Boc group in an one pot synthesis (Sakaitani *et al.*, 1988) with the structure 3-(4-((benzyloxycarbonyl)(methyl)amino)-2-methyl-5-oxo-1-phenyl-2,5-dihydro-1*H*-pyrazol-3-yl)propanoic acid, a ringclosure to sixmembered ring was formed. For further investigation, we omitted the step of conversion and performed a direct clevage reaction (Greene & Wuts, 1999) leading to the title compound.

The pyrazolone ring of the anellated ringsystem is oriented at a dihedral angle of 36.05 (7)° with respect to the phenyl ring. The methyl group (C17) shows a distance of 0.864 (2) Å to the least square plane of the pyrazolone ring system.

### Experimental

The compound was prepared by palladium catalzyed cleaverage of a N-Cbz protected amine (Greene & Wuts, 1999). 3-(4-((Benzyloxycarbonyl)(methyl)amino)-2-methyl-5-oxo-1-phenyl-2,5-dihydro-1*H*-pyrazol-3-yl)propanoic acid (0.74 g, 1.81 mmol) and palladium on activated carbon (10%, 0.19 g, 0.18 mmol) were dissolved in 40 ml ethyl acetate under a hydrogen atmosphere (1 atm). The mixture was stirred for 12 h with regular TLC monitoring, then filtered and concentrated. The resulting residue was purified by flash chromatography (SiO_2_, ethyl acetate/isopropyl alcohol 1:1). Crystals of the title compound were obtained by slow evaporation of ethanol at room temperature.

### Refinement

In the absence of anomalous scatterers, Friedel pairs were merged. Hydrogen atoms   were placed at calculated positions with C—H = 0.95 Å (aromatic) or 0.98–0.99 Å (*sp*^3^ C-atom). All H atoms were refined in the riding-model approximation with isotropic displacement parameters  set at 1.2–1.5 times of the *U*_eq_ of the parent atom.

### Crystal data

**Table:**

C_14_H_15_N_3_O_2_	*F*(000) = 544
*M**_r_* = 257.29	*D*_x_ = 1.374 Mg m^−^^3^
Monoclinic, *C**c*	Mo *K*α radiation, λ = 0.71073 Å
Hall symbol: C -2yc	Cell parameters from 6605 reflections
*a* = 8.9721 (7) Å	θ = 2.8–29.3°
*b* = 21.7653 (19) Å	µ = 0.10 mm^−^^1^
*c* = 7.3725 (5) Å	*T* = 193 K
β = 120.214 (5)°	Plate, colourless
*V* = 1244.12 (17) Å^3^	0.45 × 0.17 × 0.16 mm
*Z* = 4	

### Data collection

**Table:**

Stoe IPDS 2T diffractometer	1541 reflections with *I* > 2σ(*I*)
Radiation source: sealed Tube	*R*_int_ = 0.036
graphite	θ_max_ = 28.9°, θ_min_ = 2.8°
Detector resolution: 6.67 pixels mm^-1^	*h* = −12→12
rotation method scans	*k* = −29→25
4103 measured reflections	*l* = −9→10
1623 independent reflections	

### Refinement

**Table:**

Refinement on *F*^2^	Primary atom site location: structure-invariant direct methods
Least-squares matrix: full	Secondary atom site location: difference Fourier map
*R*[*F*^2^ > 2σ(*F*^2^)] = 0.027	Hydrogen site location: inferred from neighbouring sites
*wR*(*F*^2^) = 0.071	H-atom parameters constrained
*S* = 1.04	*w* = 1/[σ^2^(*F*_o_^2^) + (0.0454*P*)^2^ + 0.1582*P*] where *P* = (*F*_o_^2^ + 2*F*_c_^2^)/3
1623 reflections	(Δ/σ)_max_ < 0.001
174 parameters	Δρ_max_ = 0.18 e Å^−^^3^
2 restraints	Δρ_min_ = −0.14 e Å^−^^3^

### Special details

**Table:**

Geometry. All e.s.d.'s (except the e.s.d. in the dihedral angle between two l.s. planes) are estimated using the full covariance matrix. The cell e.s.d.'s are taken into account individually in the estimation of e.s.d.'s in distances, angles and torsion angles; correlations between e.s.d.'s in cell parameters are only used when they are defined by crystal symmetry. An approximate (isotropic) treatment of cell e.s.d.'s is used for estimating e.s.d.'s involving l.s. planes.
Refinement. Refinement of *F*^2^ against ALL reflections. The weighted *R*-factor *wR* and goodness of fit *S* are based on *F*^2^, conventional *R*-factors *R* are based on *F*, with *F* set to zero for negative *F*^2^. The threshold expression of *F*^2^ > σ(*F*^2^) is used only for calculating *R*-factors(gt) *etc*. and is not relevant to the choice of reflections for refinement. *R*-factors based on *F*^2^ are statistically about twice as large as those based on *F*, and *R*- factors based on ALL data will be even larger.

### Fractional atomic coordinates and isotropic or equivalent isotropic displacement parameters (Å^2^)

**Table:**

	*x*	*y*	*z*	*U*_iso_*/*U*_eq_	
N1	0.35428 (16)	0.22736 (6)	0.2232 (2)	0.0248 (3)	
C2	0.31867 (18)	0.27099 (7)	0.3359 (2)	0.0250 (3)	
C3	0.36758 (18)	0.32872 (6)	0.2826 (2)	0.0241 (3)	
C4	0.43857 (18)	0.31670 (7)	0.1635 (2)	0.0240 (3)	
N5	0.44486 (16)	0.25431 (6)	0.1332 (2)	0.0239 (2)	
C6	0.37458 (19)	0.16362 (7)	0.2688 (2)	0.0245 (3)	
C7	0.2491 (2)	0.13330 (7)	0.2930 (3)	0.0305 (3)	
H7	0.1517	0.1550	0.2782	0.037*	
C8	0.2679 (3)	0.07098 (8)	0.3392 (3)	0.0386 (4)	
H8	0.1846	0.0503	0.3602	0.046*	
C9	0.4070 (3)	0.03872 (8)	0.3550 (3)	0.0420 (4)	
H9	0.4181	−0.0040	0.3852	0.050*	
C10	0.5303 (2)	0.06878 (8)	0.3266 (3)	0.0373 (4)	
H10	0.6246	0.0465	0.3351	0.045*	
C11	0.5156 (2)	0.13149 (8)	0.2858 (3)	0.0301 (3)	
H11	0.6012	0.1524	0.2696	0.036*	
O12	0.26349 (16)	0.25874 (6)	0.45386 (19)	0.0330 (3)	
N13	0.35152 (17)	0.38830 (6)	0.3448 (2)	0.0291 (3)	
C14	0.4310 (2)	0.43628 (7)	0.3067 (3)	0.0311 (3)	
C15	0.5584 (2)	0.41980 (8)	0.2363 (3)	0.0328 (3)	
H15A	0.6707	0.4100	0.3621	0.039*	
H15B	0.5757	0.4563	0.1690	0.039*	
C16	0.5053 (2)	0.36558 (7)	0.0828 (3)	0.0290 (3)	
H16A	0.4149	0.3785	−0.0594	0.035*	
H16B	0.6059	0.3503	0.0748	0.035*	
C17	0.3917 (2)	0.23369 (8)	−0.0807 (2)	0.0291 (3)	
H17A	0.2717	0.2458	−0.1759	0.044*	
H17B	0.4018	0.1889	−0.0821	0.044*	
H17C	0.4662	0.2527	−0.1267	0.044*	
C18	0.2290 (3)	0.40112 (8)	0.4156 (3)	0.0394 (4)	
H18A	0.2698	0.4362	0.5119	0.059*	
H18B	0.2191	0.3650	0.4883	0.059*	
H18C	0.1159	0.4107	0.2942	0.059*	
O19	0.4061 (2)	0.48961 (6)	0.3370 (2)	0.0423 (3)	

### Atomic displacement parameters (Å^2^)

**Table:**

	*U*^11^	*U*^22^	*U*^33^	*U*^12^	*U*^13^	*U*^23^
N1	0.0286 (6)	0.0233 (6)	0.0300 (6)	−0.0013 (4)	0.0203 (5)	0.0012 (5)
C2	0.0246 (6)	0.0261 (7)	0.0255 (6)	0.0001 (5)	0.0136 (5)	0.0008 (5)
C3	0.0255 (6)	0.0227 (6)	0.0252 (7)	−0.0010 (5)	0.0136 (5)	0.0018 (5)
C4	0.0232 (6)	0.0254 (7)	0.0241 (6)	−0.0015 (5)	0.0124 (5)	0.0023 (5)
N5	0.0259 (5)	0.0252 (6)	0.0259 (6)	−0.0023 (4)	0.0171 (5)	0.0005 (5)
C6	0.0281 (6)	0.0216 (6)	0.0244 (6)	−0.0030 (5)	0.0136 (5)	−0.0020 (5)
C7	0.0339 (8)	0.0282 (8)	0.0337 (8)	−0.0062 (6)	0.0203 (6)	−0.0040 (6)
C8	0.0523 (10)	0.0289 (8)	0.0423 (10)	−0.0126 (7)	0.0295 (8)	−0.0047 (7)
C9	0.0576 (11)	0.0207 (7)	0.0462 (10)	−0.0034 (7)	0.0249 (8)	0.0007 (7)
C10	0.0389 (8)	0.0272 (8)	0.0416 (10)	0.0044 (6)	0.0173 (7)	−0.0011 (6)
C11	0.0272 (7)	0.0269 (7)	0.0333 (8)	−0.0022 (5)	0.0130 (6)	−0.0022 (6)
O12	0.0435 (6)	0.0324 (6)	0.0363 (6)	−0.0002 (5)	0.0299 (5)	0.0015 (5)
N13	0.0343 (6)	0.0240 (6)	0.0316 (6)	0.0003 (5)	0.0185 (5)	−0.0012 (5)
C14	0.0378 (8)	0.0243 (7)	0.0265 (7)	−0.0023 (6)	0.0127 (6)	0.0009 (6)
C15	0.0341 (7)	0.0276 (7)	0.0346 (8)	−0.0066 (6)	0.0157 (6)	0.0040 (6)
C16	0.0305 (7)	0.0293 (7)	0.0297 (7)	−0.0030 (5)	0.0170 (6)	0.0053 (6)
C17	0.0277 (7)	0.0353 (8)	0.0270 (7)	−0.0014 (6)	0.0159 (6)	−0.0038 (6)
C18	0.0490 (10)	0.0332 (8)	0.0491 (10)	0.0044 (7)	0.0343 (9)	−0.0036 (7)
O19	0.0596 (8)	0.0229 (6)	0.0438 (7)	−0.0010 (5)	0.0255 (6)	−0.0012 (5)

### Geometric parameters (Å, °)

**Table:**

N1—C2	1.4009 (19)	C10—C11	1.390 (2)
N1—N5	1.4098 (16)	C10—H10	0.9500
N1—C6	1.4173 (18)	C11—H11	0.9500
C2—O12	1.2269 (18)	N13—C14	1.371 (2)
C2—C3	1.449 (2)	N13—C18	1.462 (2)
C3—C4	1.344 (2)	C14—O19	1.224 (2)
C3—N13	1.4069 (19)	C14—C15	1.517 (3)
C4—N5	1.3820 (19)	C15—C16	1.536 (2)
C4—C16	1.485 (2)	C15—H15A	0.9900
N5—C17	1.4691 (19)	C15—H15B	0.9900
C6—C7	1.392 (2)	C16—H16A	0.9900
C6—C11	1.395 (2)	C16—H16B	0.9900
C7—C8	1.388 (2)	C17—H17A	0.9800
C7—H7	0.9500	C17—H17B	0.9800
C8—C9	1.385 (3)	C17—H17C	0.9800
C8—H8	0.9500	C18—H18A	0.9800
C9—C10	1.389 (3)	C18—H18B	0.9800
C9—H9	0.9500	C18—H18C	0.9800
			
C2—N1—N5	110.78 (12)	C6—C11—H11	120.2
C2—N1—C6	124.29 (12)	C14—N13—C3	119.08 (13)
N5—N1—C6	118.88 (12)	C14—N13—C18	119.34 (14)
O12—C2—N1	124.53 (14)	C3—N13—C18	120.76 (13)
O12—C2—C3	131.78 (14)	O19—C14—N13	121.55 (16)
N1—C2—C3	103.65 (12)	O19—C14—C15	121.68 (15)
C4—C3—N13	123.53 (13)	N13—C14—C15	116.69 (14)
C4—C3—C2	108.44 (13)	C14—C15—C16	115.22 (13)
N13—C3—C2	128.01 (13)	C14—C15—H15A	108.5
C3—C4—N5	111.62 (12)	C16—C15—H15A	108.5
C3—C4—C16	122.80 (14)	C14—C15—H15B	108.5
N5—C4—C16	125.56 (13)	C16—C15—H15B	108.5
C4—N5—N1	104.62 (11)	H15A—C15—H15B	107.5
C4—N5—C17	117.15 (13)	C4—C16—C15	107.06 (13)
N1—N5—C17	115.18 (12)	C4—C16—H16A	110.3
C7—C6—C11	120.43 (14)	C15—C16—H16A	110.3
C7—C6—N1	118.59 (14)	C4—C16—H16B	110.3
C11—C6—N1	120.98 (13)	C15—C16—H16B	110.3
C8—C7—C6	119.23 (16)	H16A—C16—H16B	108.6
C8—C7—H7	120.4	N5—C17—H17A	109.5
C6—C7—H7	120.4	N5—C17—H17B	109.5
C9—C8—C7	120.61 (16)	H17A—C17—H17B	109.5
C9—C8—H8	119.7	N5—C17—H17C	109.5
C7—C8—H8	119.7	H17A—C17—H17C	109.5
C8—C9—C10	120.07 (16)	H17B—C17—H17C	109.5
C8—C9—H9	120.0	N13—C18—H18A	109.5
C10—C9—H9	120.0	N13—C18—H18B	109.5
C9—C10—C11	119.95 (16)	H18A—C18—H18B	109.5
C9—C10—H10	120.0	N13—C18—H18C	109.5
C11—C10—H10	120.0	H18A—C18—H18C	109.5
C10—C11—C6	119.67 (15)	H18B—C18—H18C	109.5
C10—C11—H11	120.2		
			
N5—N1—C2—O12	−169.21 (15)	N5—N1—C6—C11	19.2 (2)
C6—N1—C2—O12	−17.1 (2)	C11—C6—C7—C8	1.5 (2)
N5—N1—C2—C3	8.97 (15)	N1—C6—C7—C8	−179.53 (16)
C6—N1—C2—C3	161.12 (13)	C6—C7—C8—C9	−1.9 (3)
O12—C2—C3—C4	173.11 (17)	C7—C8—C9—C10	0.7 (3)
N1—C2—C3—C4	−4.88 (15)	C8—C9—C10—C11	1.1 (3)
O12—C2—C3—N13	−5.2 (3)	C9—C10—C11—C6	−1.5 (3)
N1—C2—C3—N13	176.85 (14)	C7—C6—C11—C10	0.2 (2)
N13—C3—C4—N5	177.41 (13)	N1—C6—C11—C10	−178.76 (16)
C2—C3—C4—N5	−0.95 (17)	C4—C3—N13—C14	−8.7 (2)
N13—C3—C4—C16	−0.9 (2)	C2—C3—N13—C14	169.31 (14)
C2—C3—C4—C16	−179.27 (13)	C4—C3—N13—C18	160.80 (16)
C3—C4—N5—N1	6.36 (17)	C2—C3—N13—C18	−21.2 (2)
C16—C4—N5—N1	−175.38 (13)	C3—N13—C14—O19	172.06 (16)
C3—C4—N5—C17	135.25 (13)	C18—N13—C14—O19	2.4 (2)
C16—C4—N5—C17	−46.5 (2)	C3—N13—C14—C15	−11.1 (2)
C2—N1—N5—C4	−9.63 (16)	C18—N13—C14—C15	179.24 (16)
C6—N1—N5—C4	−163.47 (12)	O19—C14—C15—C16	−143.95 (16)
C2—N1—N5—C17	−139.69 (13)	N13—C14—C15—C16	39.2 (2)
C6—N1—N5—C17	66.46 (18)	C3—C4—C16—C15	26.93 (19)
C2—N1—C6—C7	50.1 (2)	N5—C4—C16—C15	−151.14 (15)
N5—N1—C6—C7	−159.82 (13)	C14—C15—C16—C4	−44.36 (18)
C2—N1—C6—C11	−130.91 (15)		
